# Massive metamaterial system-loaded MIMO antenna array for 5G base stations

**DOI:** 10.1038/s41598-022-18329-y

**Published:** 2022-08-22

**Authors:** Samir Salem Al-Bawri, Mohammad Tariqul Islam, Md Shabiul Islam, Mandeep Jit Singh, Haitham Alsaif

**Affiliations:** 1 Space Science Centre, Climate Change Institute, Universiti Kebangsaan Malaysia (UKM), 43600 Bangi, Malaysia; 2 Department of Electrical, Electronic and Systems Engineering, Faculty of Engineering and Built Environment, Universiti Kebangsaan Malaysia, UKM, 43600 Bangi, Selangor Malaysia; 3 Faculty of Engineering, Multimedia University, Persiaran Multimedia, 63100 Cyberjaya, Selangor Malaysia; 4 Electrical Engineering Department, College of Engineering, University of Ha’il, Ha’il, 81481 Saudi Arabia

**Keywords:** Electrical and electronic engineering, Materials for devices

## Abstract

An integrated massive multiple-input multiple-output (mMIMO) antenna system loaded with metamaterial (MTM) is proposed in this article for fifth-generation (5G) applications. Besides, achievement of duple negative (DNG) characteristics using a proposed compact complementary split-ring resonator (SRR), a broad epsilon negative metamaterial (ENG) with more than 1 GHz bandwidth (BW), and near-zero refractive index (NZRI) features are presented. The proposed mMIMO antenna consists of eight subarrays with three layers that operate in the 5G mind band at 3.5 GHz (3.40–3.65 GHz) with high port isolation between adjacent antenna elements compared to an antenna that does not use MTM. Each subarray has two patches on the top layer, while the middle and bottom layers have two categories of full and partial ground plans, respectively. Simulated, produced, and tested are 32 elements with a total volume of 184 × 340 × 1.575 mm^3^. The measured findings reveal that the sub-6 antenna has a better than 10 dB reflection coefficient (S11), a lower than 35 dB isolation, and a peak gain of 10.6 dBi for each subarray. Furthermore, the recommended antenna loaded with MTM has demonstrated good MIMO performance with an ECC of less than 0.0001, total efficiencies of more than 90%, more than 300 MHz bandwidth, and an overall gain of 19.5 dBi.

## Introduction

Wireless communication systems have seen an exponential development in recent years, and this scenario leads to continue with highly demanded of sophisticated technologies. For instant, higher transmission data rate and shorter latency with increment in channel capacity are the critical parameters that must be improved significantly to satisfy the requirement of future mid-band fifth-generation (5G) wireless systems below 6 GHz. For this, massive MIMO technology is one of the potential solutions^1–3^, which can simultaneously support more users, offers improved diversity, and multiplexing, as well as enables a significant enhancement in energy-efficient systems. Massive MIMO operation has been studied widely based on homogeneous arrays and omnidirectional patterns^4–6^. However, the influence of the directional antenna gain pattern on the performance of mMIMO system has been neglected in most of these studies.

5G MIMO antenna systems have been reported for either the single or dual operational bands^7–9^. Recently, three working bands of 5G New Radio (NR) have been started by Generation Partnership Project (3GPP)^10^; these bands inholding the mid-band application at a span range of (3.3–3.8 GHz), (3.3–4.2 GHz), and (4.4–5.0 GHz) which represent N78, N77 and N79 respectively. Besides, every country can choose its own 5G demanded bands, as mentioned above. For instant, China has been officially declared to utilize two bands at (3.3–3.6 GHz) and (4.8–5.0 GHz)^11^, although the frequency band from 3.4 to 3.8 GHz has been decided by European Union (EU) for the 5G application^12^. Consequently, to cover the aforementioned 5G operating bands for mobility reasons, a specific MIMO antenna system must be developed to cover the desired 5G N77/N78/N79 bands, which is not addressed by the designs proposed in Refs.^13,14^.

Designing MIMO antennas with high isolation between elements of the antenna, low cost, less usage of energy, small size, and being lightweight is often a challenging task. However, one of the drawbacks of antenna performance is the narrow bandwidth, which restricts the usage of new wireless systems. To avoid these challenges, several methods have been sophisticated recently. For example, the reactive impedance surface (RIS) method^15^ can be used to improve antenna radiation and bandwidth characteristics by tuning the RIS between electric (PEC) and magnetic (PMC) conductors and surfaces. In addition, the overall antenna size can be reduced. The antenna performance is greatly improved in Ref.^16^ by using a two-dimensional left-handed metamaterial (LHM) design on the dielectric substrate's top (patch) and bottom (ground) sides. This method generates capacitive-inductive features due to the coupling between the designed patch and bottom plane configuration, which creates a backward traveling wave. However, a periodic structure on the ground plan is applied for a passive antenna before testing for temperature sensing, as offered in Ref.^17^. These bottom based surface layers allow a considerable improvement in the antenna size and bandwidth characteristics.

Metamaterial (MTM), as an artificial medium, has several unusual features, such as negative (refractive index, permeability, and permittivity), making it appropriate for a variety of applications, including absorber^18^, bio sensing^19^, microwave imaging^20^, antennas^21^, metamaterial coding^22^, metamaterial lensing^23^, terahertz metamaterial^24^, and microwave devices like GPS5, WiMAX^25^.

In Ref.^26^, there is a negative index metamaterial made up of extended beams enclosed to a plate. A 3 dimensions acoustic MTM is examined in Ref.^27^ which can be used to create a bandgap at the location of deep sound attenuation which can be employed as an acoustic filter for noise cancellation. All of these metamaterials’ features have appeared at specified frequency bands of interest, dependent on the geometrical arrangement in the array and the structure’s fixed composition. As a result, there is a growing interest in MTMs that emphasize numerous operating frequencies that are tuned by diverse stimuli such as electrical, mechanical, or optical signals. In addition, certain MTMs and resonators for diverse applications and property analyses are described in Ref.^28^. In Ref.^29^, a hexagonal Gap coupled split-ring resonator-based MTM with a size of 10 × 10 mm^2^ that covers the S and X bands is described. MTM with a concentric ring-based resonator, on the other hand, is demonstrated in Ref.^30^, which demonstrates a single negative characteristic with dual resonances at 13.9 GHz and 27.5 GHz to improve the microstrip transmission line performance. In Ref.^31^, a triple-band response is reported for an open delta shaped ENG MTM. Furthermore, for microwave applications at S, C, and X-band, a pi-shaped complementary split-ring resonator (CSRR) associated with metal inclusion is created and described in Ref.^32^.

MTM unit cell was verified to increase antennas' performance in terms of gain, isolation, bandwidth, radiation patterns, …etc. due to its ability to perturb the current distribution patch along with the antenna radiator. In contrast, negative real value properties have occurred on the achieved refractive index (NRI) as well as both permeability (µ) and permittivity (ɛ)^33,34^. However, a MTM with a near-zero refractive index (NZRI) characteristic has been studied to improve the overall performance of the antenna in specific bands, including S, C, and X bands^35^. In addition, various types of MTMs are used to minimize the coupling between array elements^36,37^. However, the previously suggested decoupling approaches are difficult to build and operate on miniaturize MIMO antenna elements. Unlike conventional antenna arrays, this research uses a series of small split-ring resonators (SRRs) as resonators and to increase the isolation between the antenna elements.

Few works offered a MIMO configuration without an array mode by using multimode at each element (beamsteering). Reference^38^ dispersed 108 elements along a nine-faced polyhedron ring operating at 2.4 GHz. This was accomplished by utilizing a developed patch to produce three modes per element: first (bandwidth of 238 MHz), second (254 MHz) modes have around 6.5 dBi gain, while the third mode of 102 MHz bandwidth has a gain of 1.21 dBi. Manteuffel and Martens^39^, on the other hand, adopted a sheet to accommodate four modes which covered a wide spectrum from 6 to 8.5 GHz, as well as an 11 × 11 array. With a very low envelope correlation coefficient, the port isolation was greater than 20 dB.

In this paper, quite high-isolation massive MIMO antenna with 32 elements that can cover 3400–3650 MHz is proposed for a future 5G base station with a measured bandwidth of 250 MHz. In addition, an analysis of a unique ENG/NZRI/DNG metamaterial unit cell is conducted to support the proposed design’s operational working principle, which is based on the epsilon negative and near-zero refractive index properties, which are designed to simultaneously improve the isolation and overall performance of the MIMO antenna system. Four compact square-shaped splatted parts make up the proposed MTM. In contrast to traditional isolation solutions, the proposed MTM-based technology allows for substantial decoupling of up to 32 dB between the proposed radiating MIMO antenna elements and small array elements with ECC 0.0001. The experimental data and the findings from the CST microwave studio have been compared, and they exhibit great agreement, demonstrating the precision of the proposed MTM, subarray, and MIMO antennas. The suggested antenna has a fractional BW of roughly 7.1% and has negligible mutual coupling. Table 1 compares the proposed MIMO antenna loaded with the proposed unique MTM to other antennas previously reported in the literature.

**Table 1:** Comparisons of the proposed performance to the published state-of-the-art performance.

Ref.	MIMO model	MIMO size (mm^3^)	Operating BW (GHz)	Isolation (dB)	ECC	Efficiency (%)	Gain (dB)	Technique
^13^	8 × 8	150 × 80 × 0.8	3.4–3.8	− 12	< 0.15	41–82	–	Using inverted π-shaped patch, along with inverted L-shaped open slot antenna
			5.15–5.925					
^40^	16 × 16	90 × 90 × 19.5	3.3–5.1	− 55	0.004	> 63	17.3	Differential feed network with vertical dotted slots cross-shaped patches has been used to increase the isolation
^41^	4 × 4	76.2 × 76.2 × 13.9	3.3–5.0	< − 30	< 0.1	> 90	> 5	To reduce mutual coupling, this design uses 3 decoupling techniques: a ferrite chock ring, an unique baffle structure, and a rectangular ring resonator
^42^	8 × 8	560 × 560 × 266	5–6	< − 20	< 0.0035	–	> 20	Wave control is achieved by altering the phase shift distribution of incident waves using a metasurface-based plane
		336 × 336 × 5.1						
^43^	2 × 2	250 × 250	3.5–4.9	28	–	90	8.8	A frequency selective surface (FSS) technique has been used between radiators
^44^	12 × 1	166 × 75 × 0.8	3.34–3.87	− 15	< 0.012	< 92	< 10	Two identical tapered microstrip line fed associate with T-shaped ground have been used to improve the bandwidth and isolation
^45^	4 × 4	200 × 200 × 32	2.8–5	− 27	–	–	7.63	Dipole elements with 2 chamfered designed aerial bow-tie in rectangular orientation fed by 2 coaxial cables are seen here
^46^	1 × 4	170 × 60 × 8	3.4–3.8	< − 25	–	–	5.5	To reduce mutual coupling, baffles loaded by a Split-ring resonator (SRR) are used
^47^	3 × 6	226.8 × 148.2 × 8	4–4.7	− 20	–	92	16	The influence of channel correlation has been investigated on indoor wideband massive MIMO to reduce the size and mutual coupling
^48^	1 × 7	140.6 × 140.6 × 1	2.85–3.1	< − 17	–	70	4	By using the theory of specific modes (TCM) compound with a modified snowflake-shaped patches
This work	4 × 8	184 × 340 × 3.15	3.4–3.65	< − 35	$\sim <$ 0.0001	> 95	> 19.5	Epsilon negative metamaterial and near-zero refractive index (ENG/ NZRI/DNG) method properties have been used for high isolation between the elements as well as to increase the gain

### Design of metamaterial unit cell

**Figure 1:**
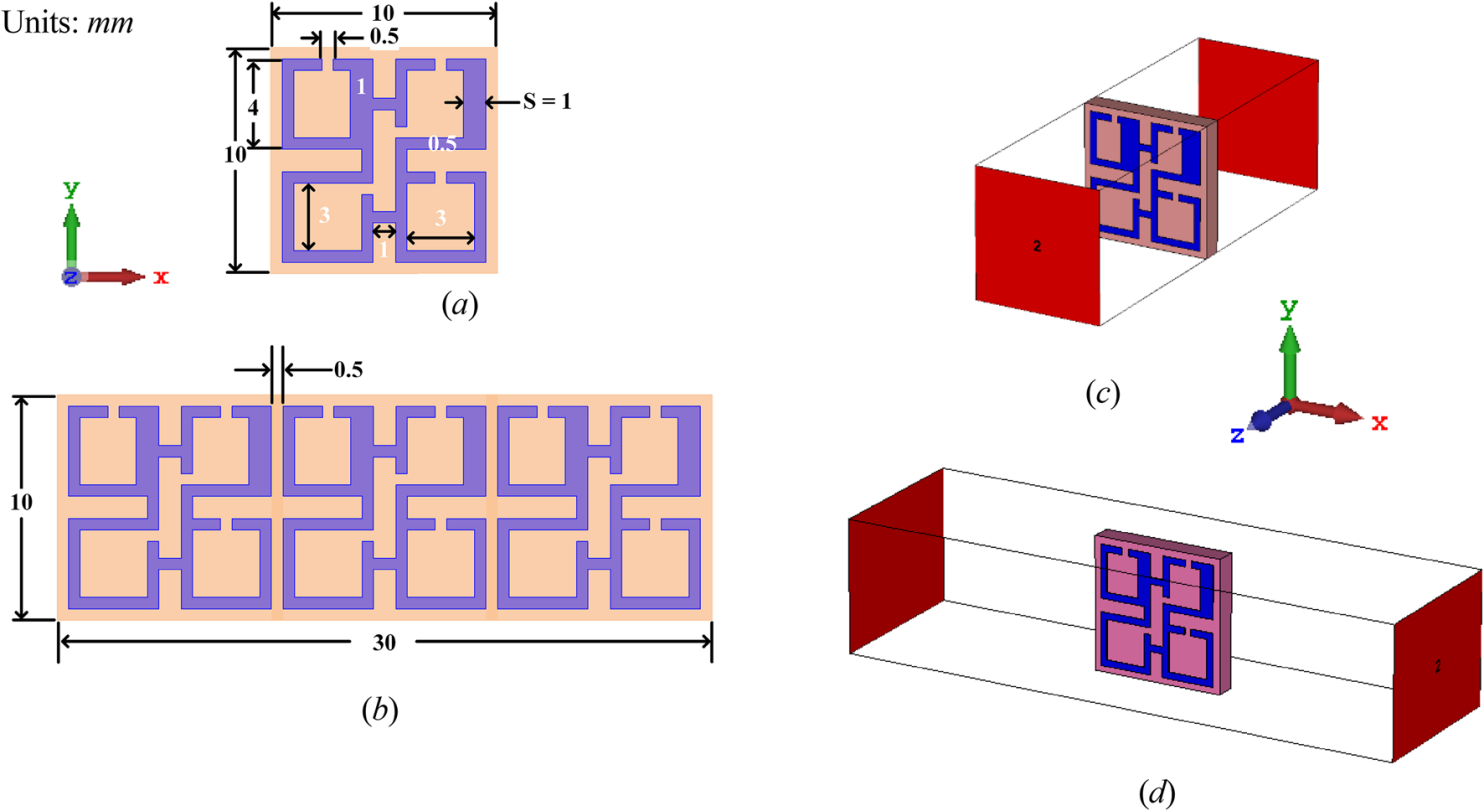
The proposed metamaterial unit cell structure: (**a**) unit cell geometry, (**b**) 3 × 1 MTM unit cell array, (**c**), simulation set up in z-axis, (**d**) simulation set up in x-axis.

Figure 1a shows the schematic view of the proposed ENG metamaterial unit cell along with its geometrical configuration parameters. It is composed of four low profile square split-ring resonators (SSRR) combined by an electrical 0.5 mm width slab and printed on the front side of Rogers 5880 substrate with a thickness of 1.575 mm, a dielectric constant εr of 2.2 and loss tangent δ of 0.0009. The proposed ENG metamaterial includes two symmetrical crescent shaped SSRR with a middle split portion on the upper arms, whereas the other two are splitted on the corner of either right or left SSRR arms. An array prototype of 1 × 3 unit cells is shown in Fig. 1b. It is created on top of the same substrate at vertical x axis-direction with 0.5 mm distance between each two units. Figure 1c characterizes the electromagnetic simulated wave propagation of the suggested ENG-DNG metamaterial design in the z-direction wherever it was positioned between 2 waveguide ports. Both perfect electric and perfect magnetic conductors (PEC, PMC) boundary conditions were applied to x-axis, and y-axis respectively. Furthermore, x-direction is also chosen to simulate the proposed MTM whereas PEC and PMC boundary conditions were utilized at z-axis, and y-axis respectively as shown in Fig. 1d.

**Figure 2:**
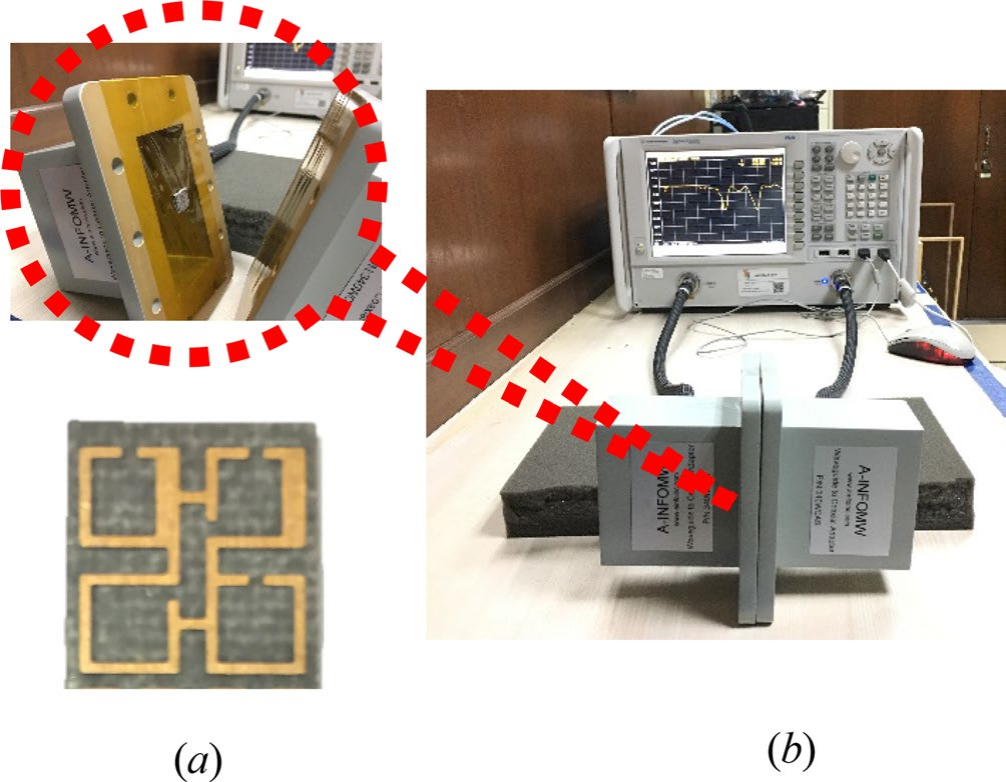
Metamaterial experimental setup: (**a**) measurement of MTM S-parameters, (**b**) unit cell with Horn antenna.

Using the normal incidences data of scattering parameters, a robust approach is used to obtain the valuable metamaterial parameters^46^. The transmission (S21) and reflection coefficients (S11) of the designed MTM unit cell are first evaluated using simulations in the frequency span from 2 to 4 GHz. Using an Agilent N5227 PNA Microwave Network Analyzer with waveguides to co-axial adapters, the S-parameters of the proposed MTM unit cell are extracted. For the appropriate frequency range, a SAR-1834031432-KF-S2-DR (1–18 GHz) waveguide was employed, and the MTM fabricated prototype has been arranged for measurements purpose between two waveguides at z-axis directions, as illustrated in Fig. 2.

### Metamaterial unit cell working principle

**Figure 3:**
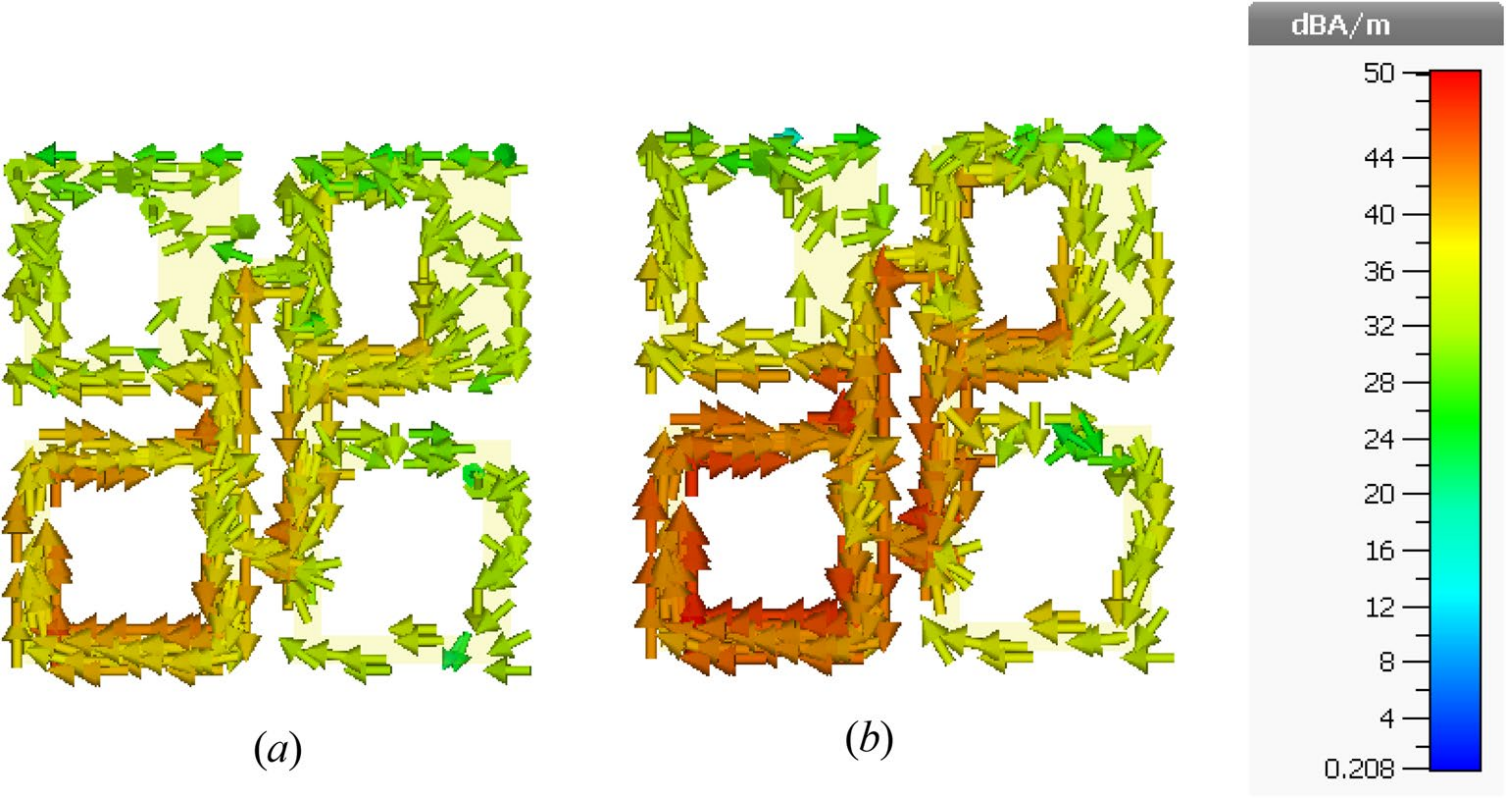
Unit cell surface current distribution at (**a**) 3.4 GHz, (**b**) 3.5 GHz.

For more understanding of the physical MTM phenomena in both electric and magnetic field zones, the surface current distribution of two selected frequencies is investigated. Figure 3a,b illustrate the suggested unit cell MTM surface current distributions at 3.4 and 3.5 GHz, respectively. The surface current density is declared by colors, whereas the arrows denote the direction of surface current distribution.

**Figure 4:**
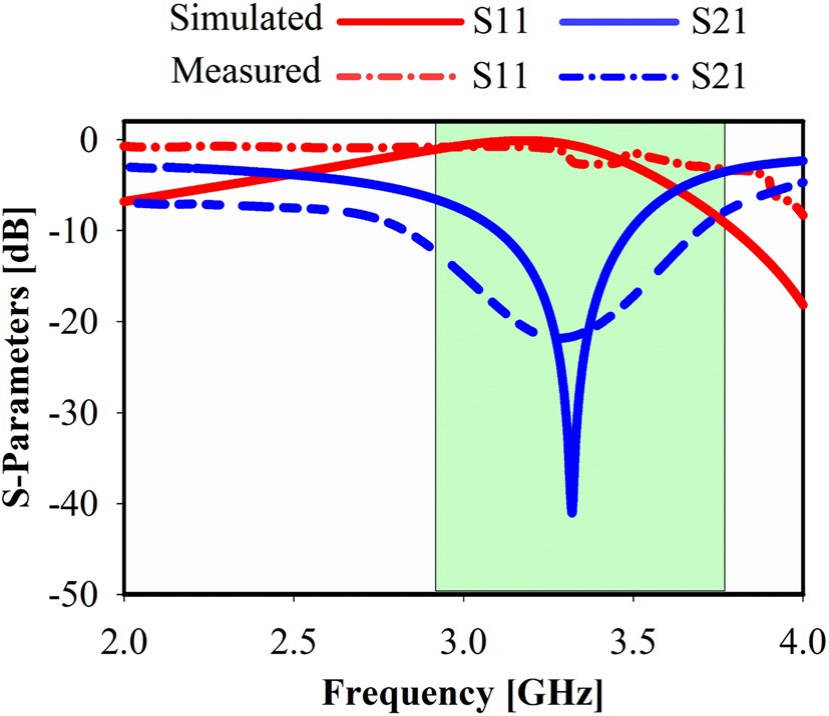
Metamaterial (MTM) s-parameters at the z-axis: measured and simulated.

At 3.4 GHz, a noticeable surface current can be seen, as shown in Fig. 3a. On the inner edge of the left bottom square-shaped part, though, the surface current is further strong and intense. Furthermore, the overall MTM unit cell structure perturbs the surface current. Although, once the current flows, opposite side orientations of the noticeable current distribution of MTM-shaped etching strips are seen, nullifying the current and forming a stopband. Though, more concentrated surface current can be clearly detected at 3.5 GHz in Fig. 3b, particularly surrounding the SSR junction, which perturbed the overall structure of the unit cell. The measured S-parameters (S21 and S11) results as well as the simulated results at z-direction are demonstrated in Fig. 4. Its illustrations show that the frequency band at the span of (3.4–3.65 GHz) is a part of the S-band and covers the mid-band of the 5G application. All split square-shaped resonators integrated with the strip line arms are considered the appropriate cause of the realized stopband operational band.

**Figure 5:**
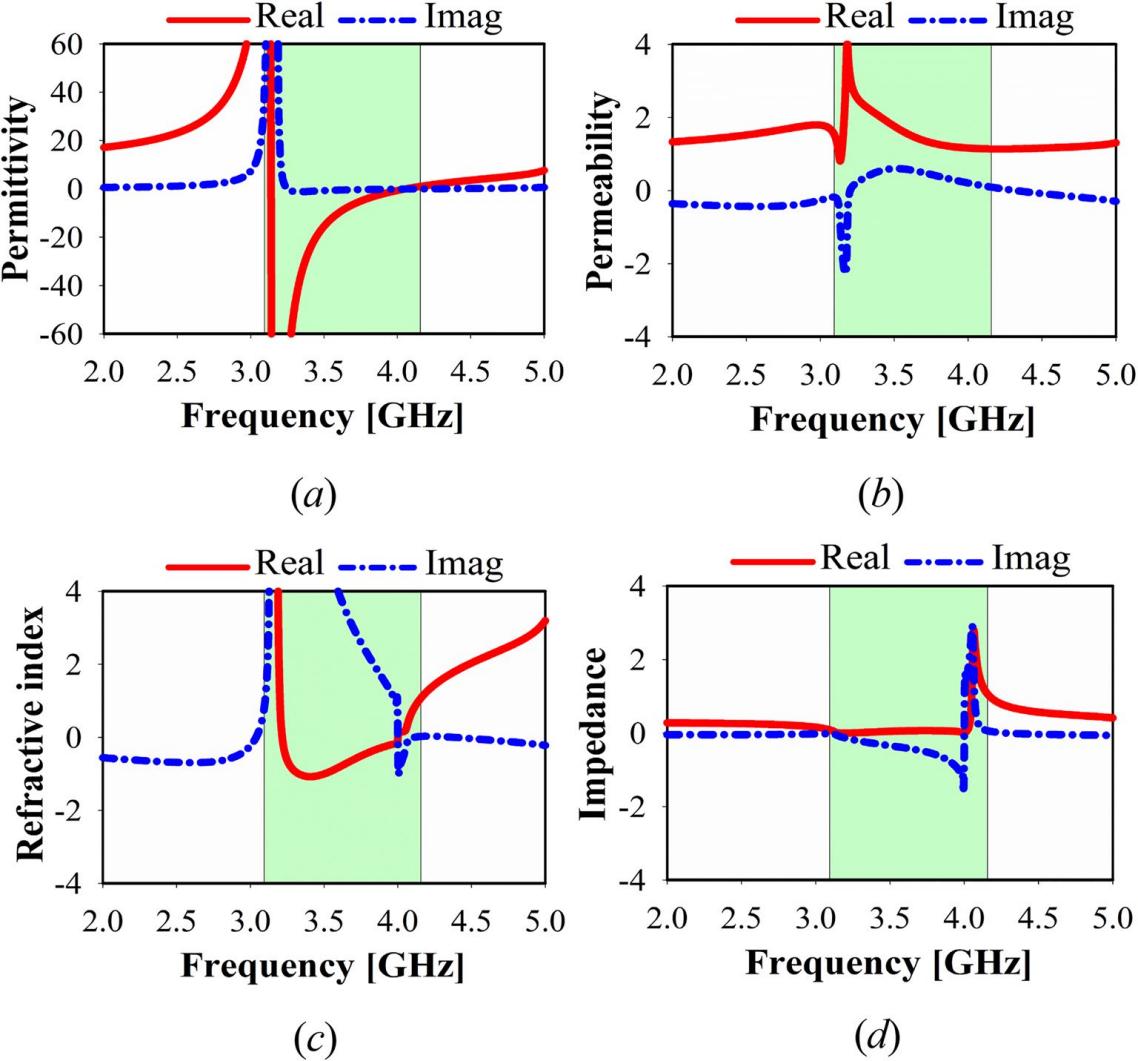
MTM, simulated results at z-axis of 1 × 1 unit cell: (**a**) permittivity, (**b**) permeability, (**c**) refractive index, (**d**) impedance.

Figure 5 shows the proposed effective parameters values of MTM. For various MTM unit cell and array configurations, these characteristics involve effective real values and imaginary parts of the realized permeability, refractive index, impedance, and permittivity. The negative indexed region for Epsilon-negative metamaterial (ENG) and near-zero refractive index metamaterial (NZRI) metamaterial is emphasized with light green color in all diagrams. An extensive negative real value of permittivity is achieved with more than 1 GHz bandwidth, as shown in Fig. 5a. Nevertheless, a NZRI property exhibits in the range of (3.1–4.2 GHz) at z-axis wave propagation, as shown in Fig. 5c. Hence, this frequency resonance band can be used for electromagnetic cloaking, high isolation, and high gain antenna design.

**Figure 6:**
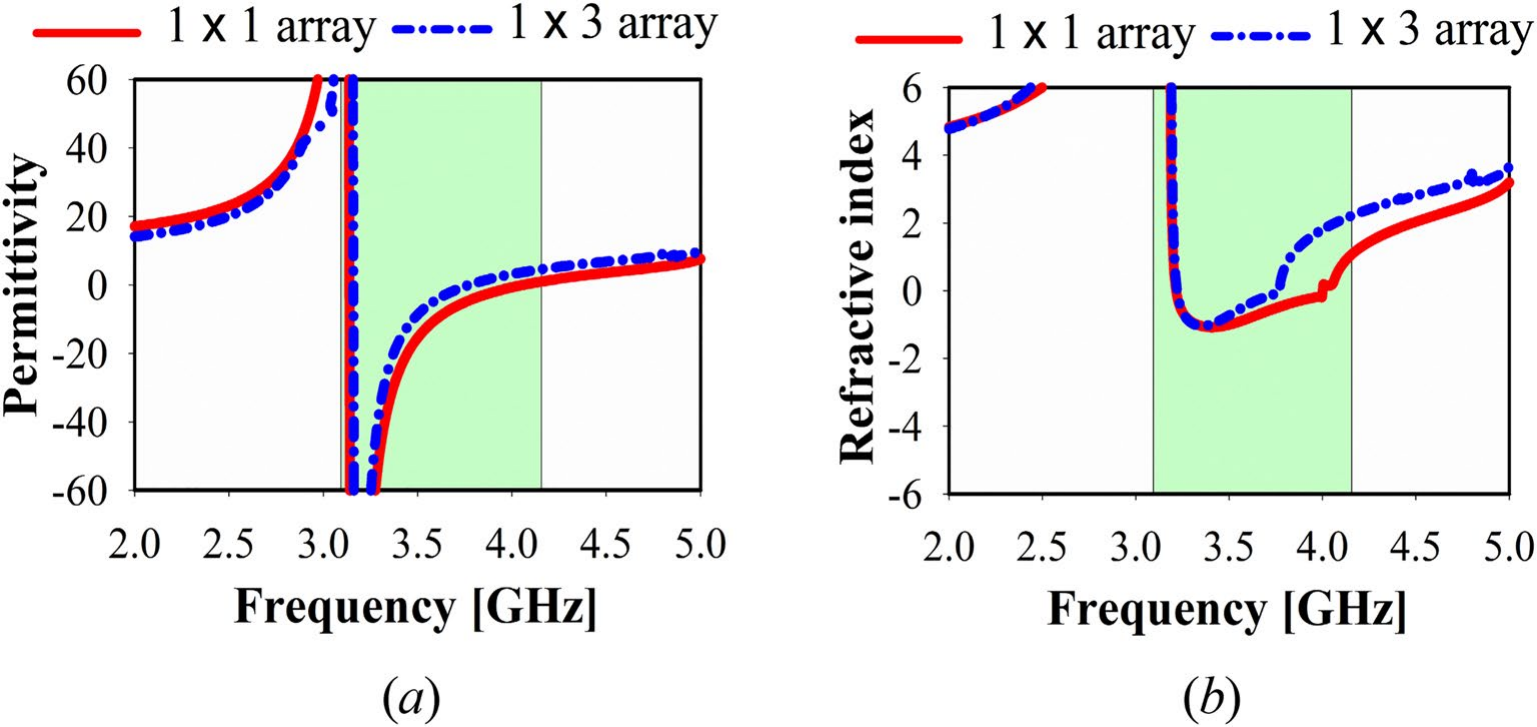
MTM, simulated results of 3 × 3 unit cell: (**a**) refractive index, (**b**) permittivity.

**Figure 7:**
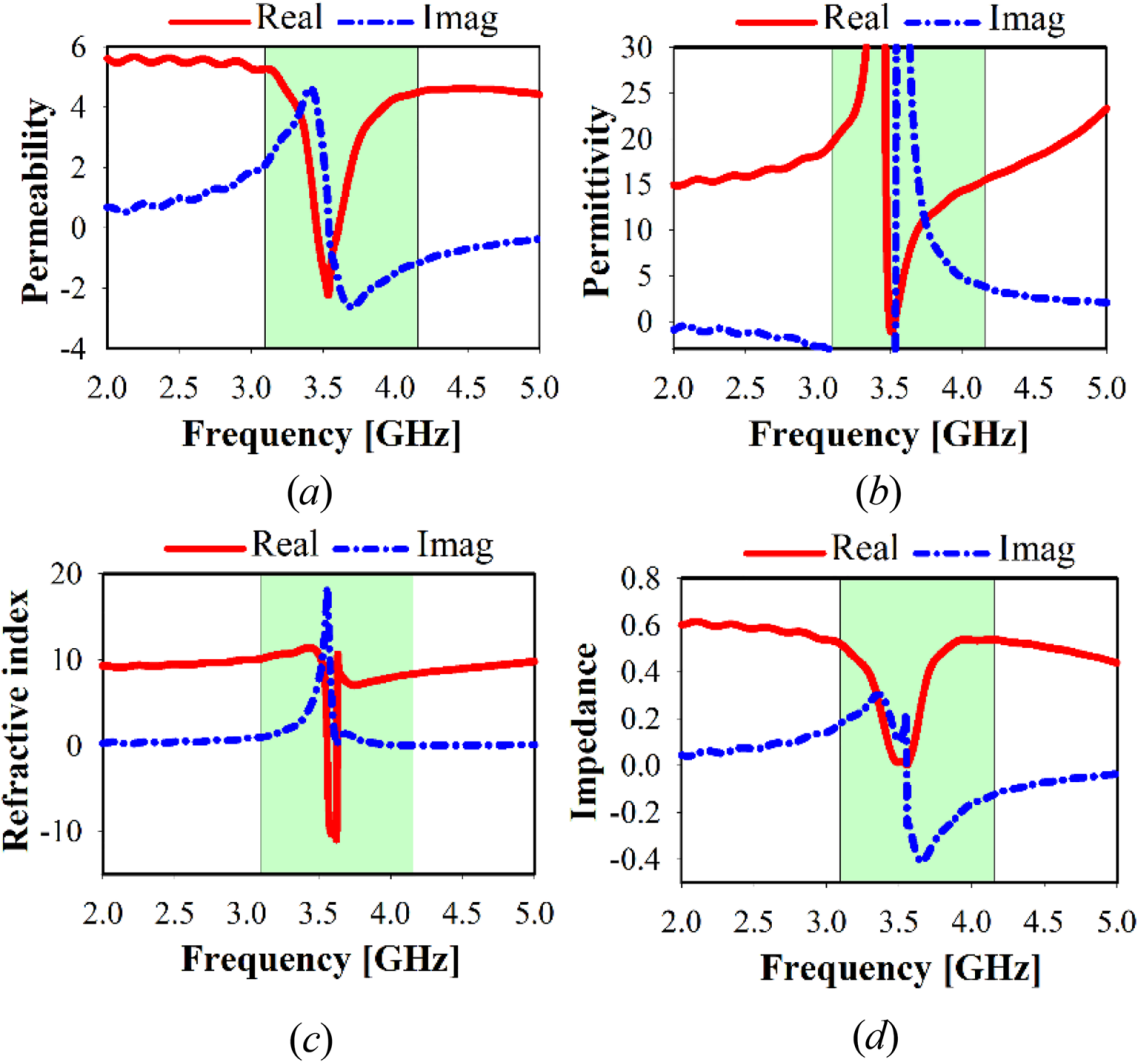
MTM, simulated results at x-axis of 1 × 1 unit cell: (**a**) permittivity, (**b**) permeability, (**c**) refractive index, (**d**) impedance.

In the z-direction, Fig. 6 illustrates the simulated relative permittivity and refractive index for the various MTM of the 1 × 1, and 1 × 3 array structures. Using one or three array unit cells, similar findings were obtained over a wide frequency range of 3–4.2 GHz. On the other hand, double negative refractive index (DNG) has been achieved along with x-axis in the frequency band (3.49–3.62 GHz) as shown in Fig. 7.

### Design of mMIMO antenna system

**Figure 8:**
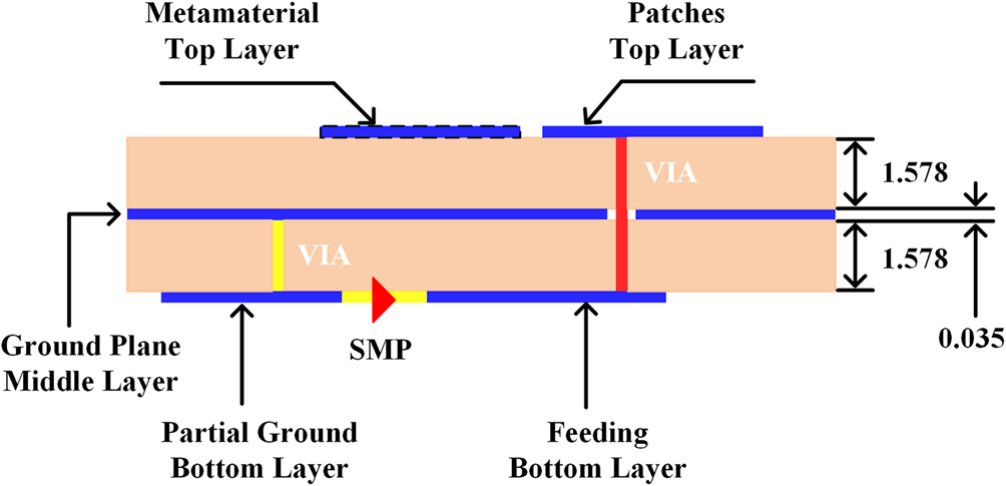
Stackup of the PCB board design displaying the layers.

**Figure 9:**
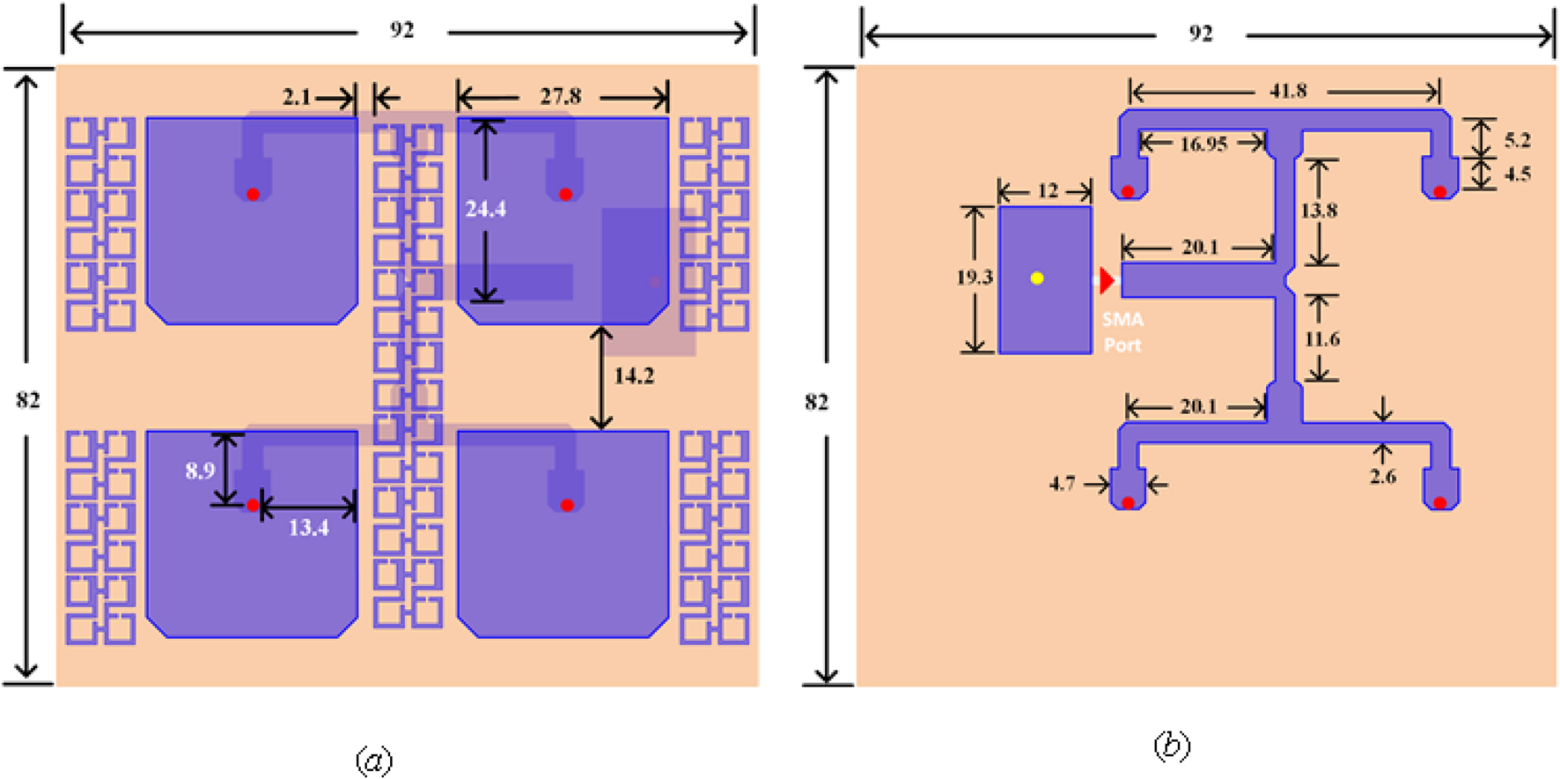
Single port (subarray), (**a**) top layer with 2 × 2 patches, (**b**) bottom layer with feeding network (all dimensions are in mm).

A subarray designed configuration consists of 2 × 2 patches feeding by a single port built on two dielectric layers of the printed circuit board (PCB), including three copper-clad laminate layers. The top layer is used for the printed patch elements. The developed feeding network (FN) is located at the bottom layer with small partial ground. The middle layer will act as an inclusive reference for both feeding network and antenna patches. Besides, 1.28 mm diameter vias are used as a probe feeding between FN and radiator elements as well as to connect both partial and full ground planes. The substrates used are Rogers 5880 with 2.2 dielectric constant, 1.575 mm thickness, and 0.0009 loss tangent. Figure 8 shows the stackup of the board design. Figure 9a,b illustrate the top and bottom layers of the single-port subarray.

**Figure 10:**
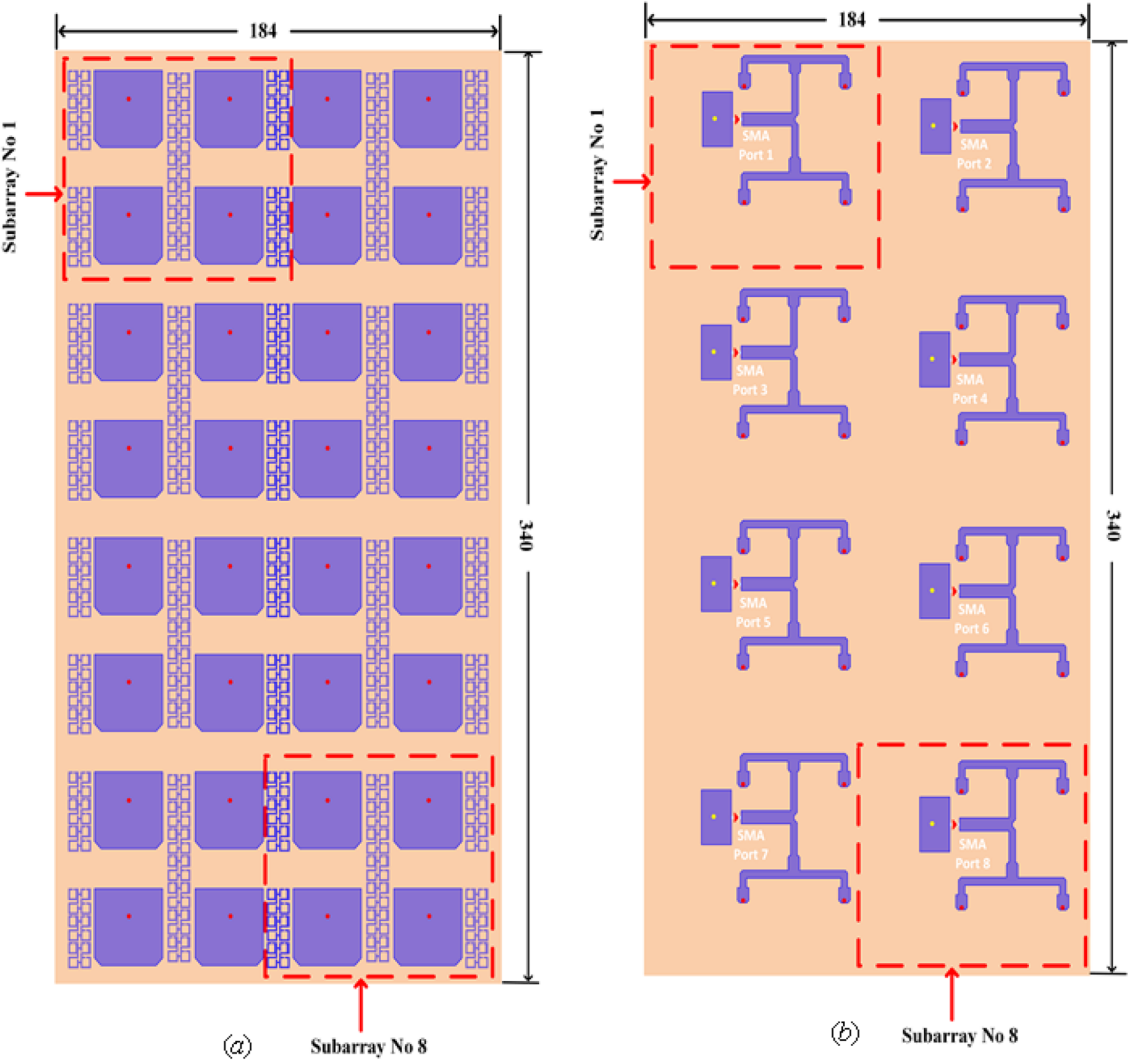
(**a**) Top view of a single side array, (**b**) single side array bottom layer.

**Figure 11:**
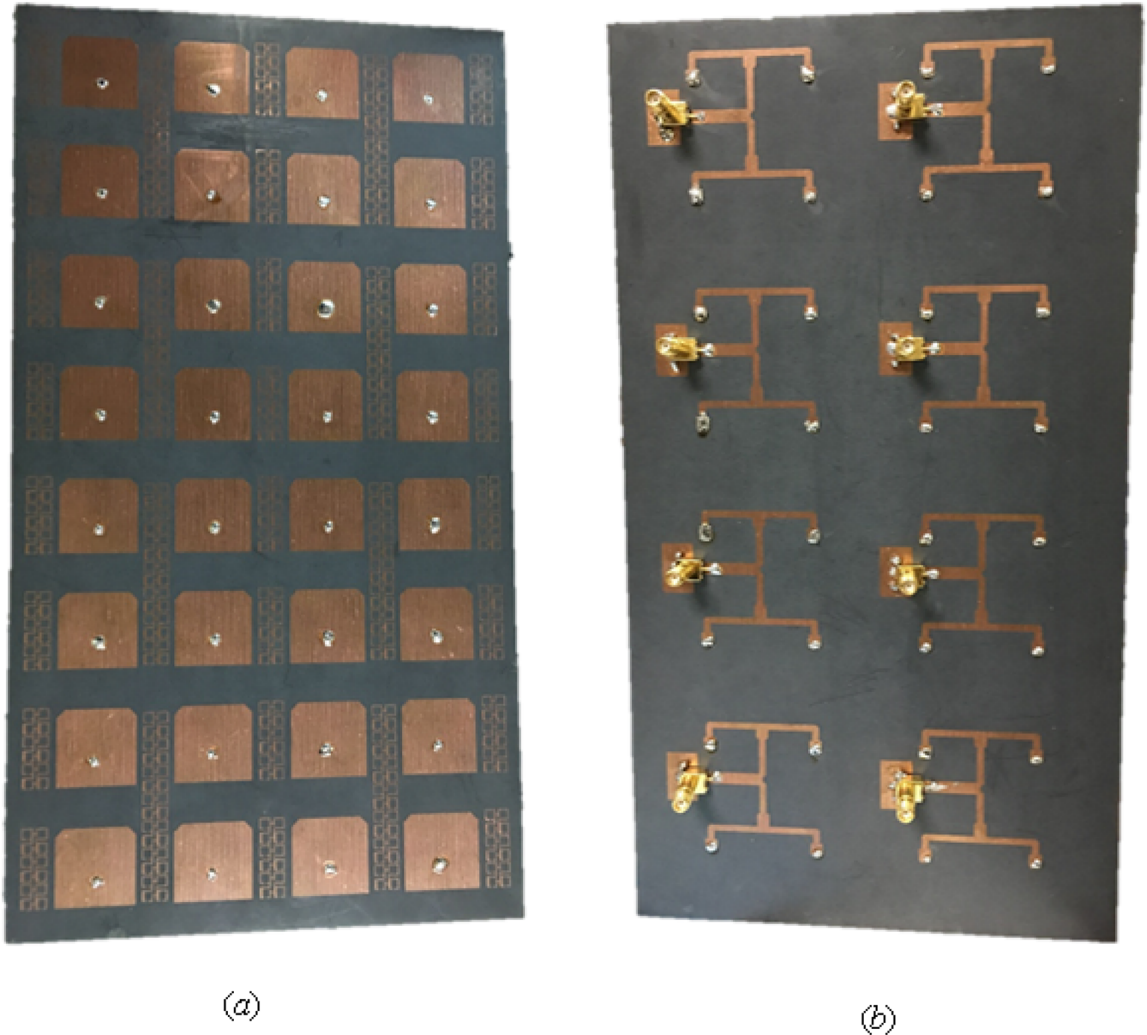
(**a**) Top view, (**b**) bottom view of fabricated single-side prototype.

The broadband side of the 8-port (32 elements) mMIMO antenna system loaded with MTM is displayed in Fig. 10. Furthermore, a 32 elements mMIMO antenna prototype is fabricated, as demonstrated in Fig. 11.

## Results and discussion

**Figure 12:**
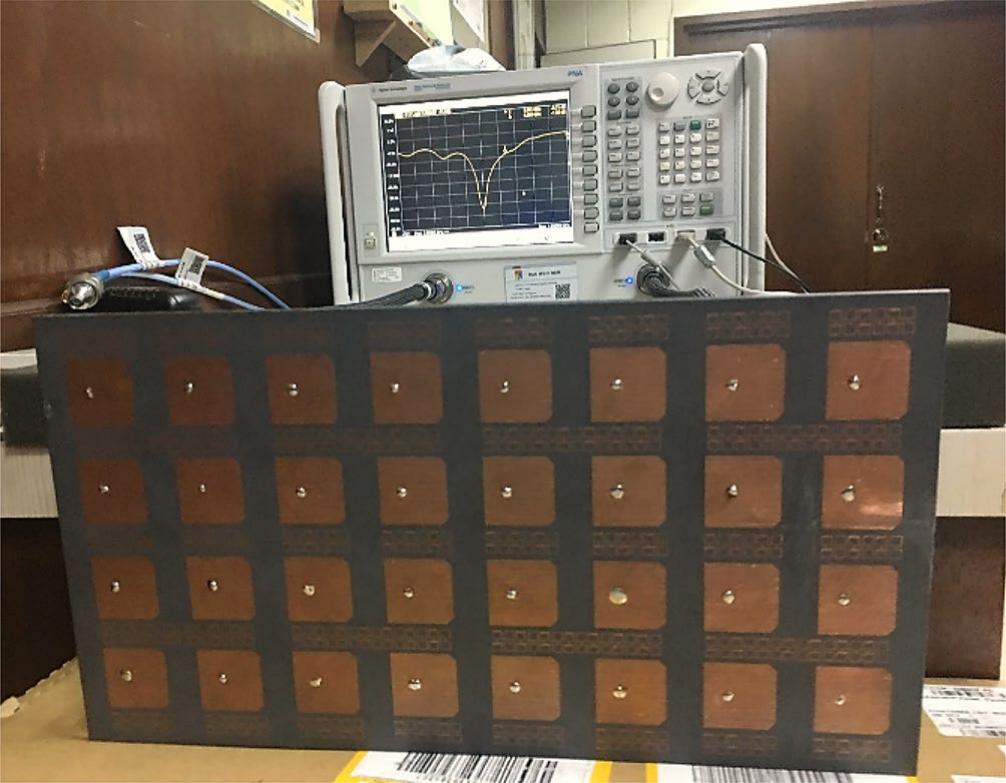
Setup for measuring the fabricating prototype.

**Figure 13:**
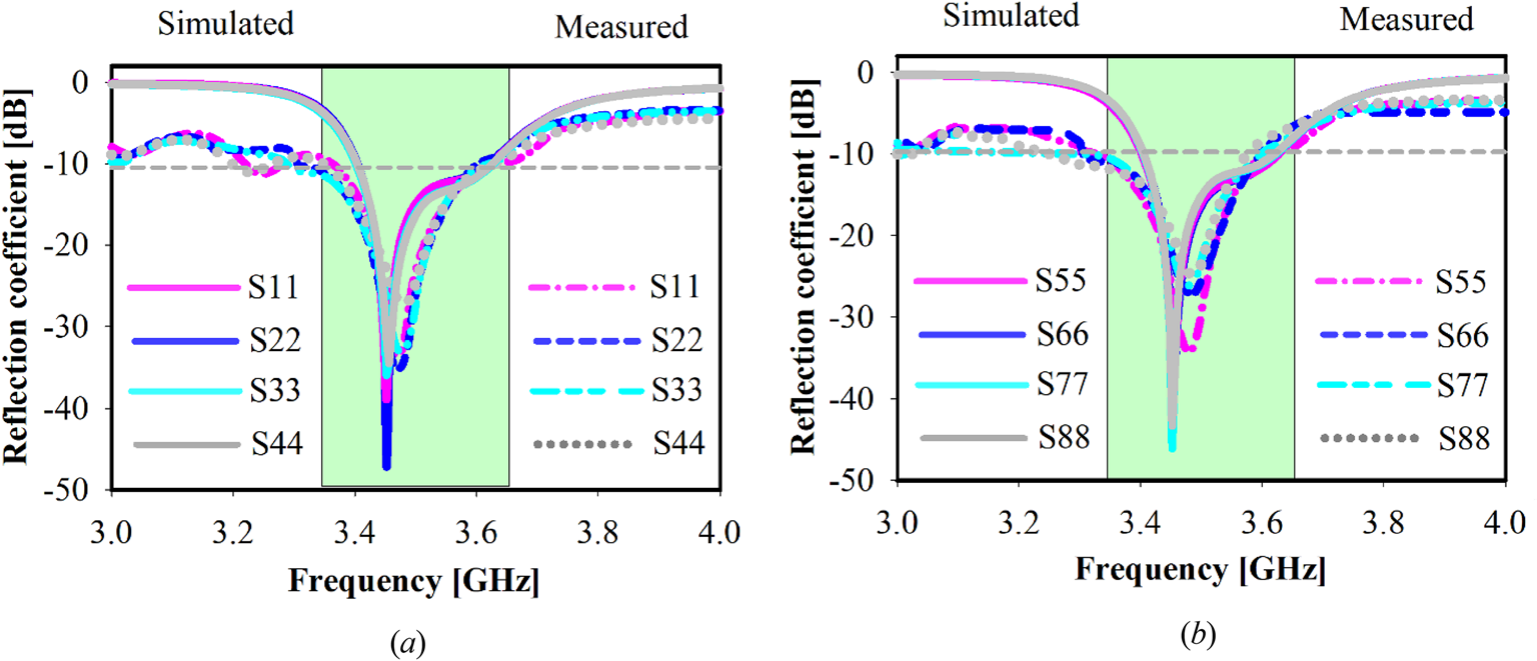
Reflection coefficient for different ports: simulated and measured, (**a**) Port 1–4, (**b**) Port 5–8.

**Figure 14:**
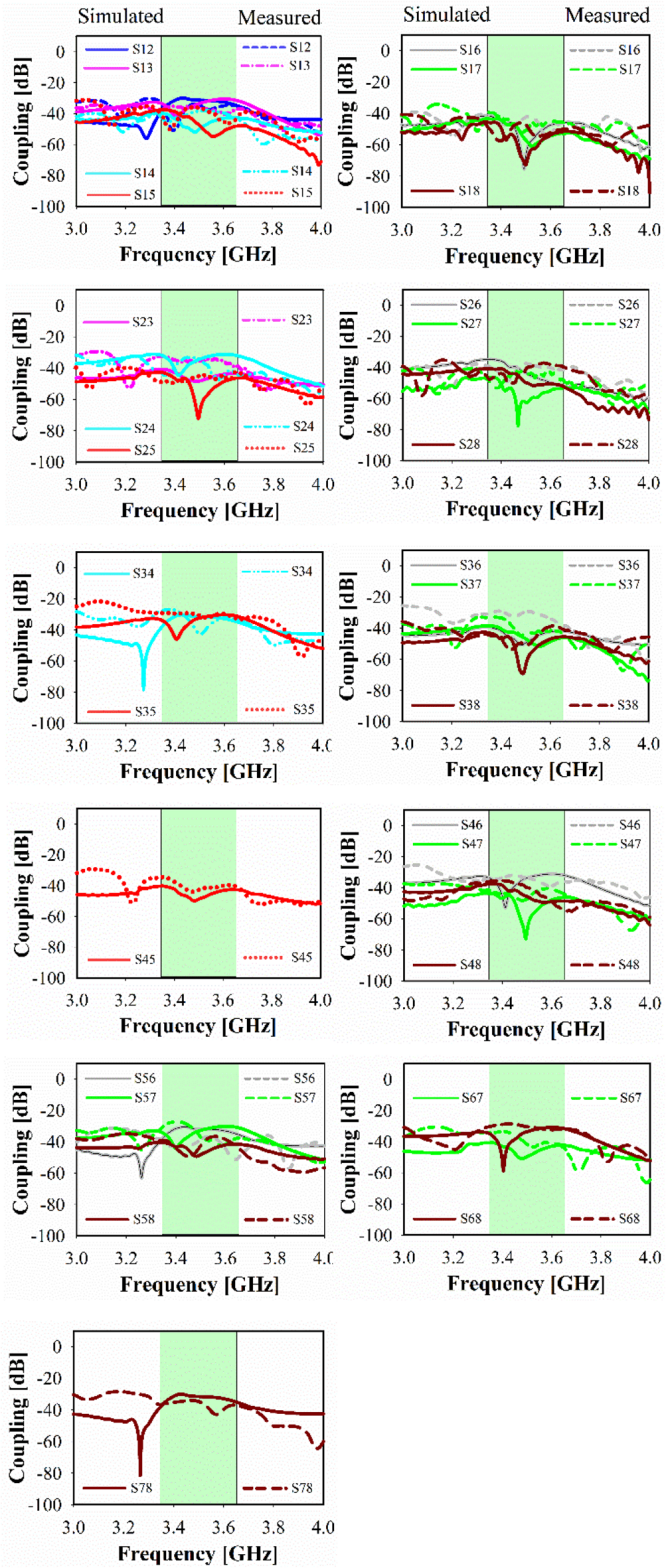
Simulated and measured coupling between each adjacent port.

Figure 12 depicts the fabricated board’s S-parameters measurement setup. Figures 13a,b show the measured and simulated reflection coefficients (S*ii*) at each port whereas (*i* = 1,2,3,4,…, 8). The simulated achieved band for each subarray is 250 MHz in the frequency span of 3.40–3.65 GHz. Around 50 MHz variation has been observed between the measured and simulated findings due to a slightly larger ground footprint was used in the simulation as well as due to the difficulties of sticking two layers. Besides large BW, high isolation is attained for every two adjacent ports as shown in Fig. 14, whereas the minimum coupling between the ports is − 32 dB in the band of interest due to the decoupling effect of the MTM, which means the radiation energy from every two close elements is coupled very weakly. As a result, the adjacent subarrays are well decoupled in the band of interest. The cross-band coupling coefficients recorded between 3 and 4 GHz are also shown.

The Envelope Correlation Coefficient is used to evaluate the proposed antenna’s MIMO diversity performance (ECC). To ensure MIMO array mode operation, the ports’ radiation patterns should be orthogonal or semi-orthogonal to one other. The ECC^49^ is the basic metric for determining the degree of correlation between distinct ports. The ECC was estimated between adjacent sub-arrays using the acquired formula and the complex electric field patterns.

1 $${\rho }_{ij}\left(e\right)=\frac{{\left|{\iint }_{4\pi }d\Omega {\overrightarrow{F}}_{i}\left(\theta ,\phi \right)\times {\overrightarrow{F}}_{j}\left(\theta ,\phi \right)\right|}^{2}}{{\iint }_{4\pi }d\Omega {\left|{\overrightarrow{F}}_{i}\left(\theta ,\phi \right)\right|}^{2}.{\iint }_{4\pi }d\Omega {\left|{\overrightarrow{F}}_{j}\left(\theta ,\phi \right)\right|}^{2}},$$

whereas ${\overrightarrow{F}}_{i}\left(\theta ,\phi \right)$ and ${\overrightarrow{F}}_{j}\left(\theta ,\phi \right)$ are 2 considered radiating elements of the antenna in far field characteristic with respect to *θ*.

**Figure 15:**
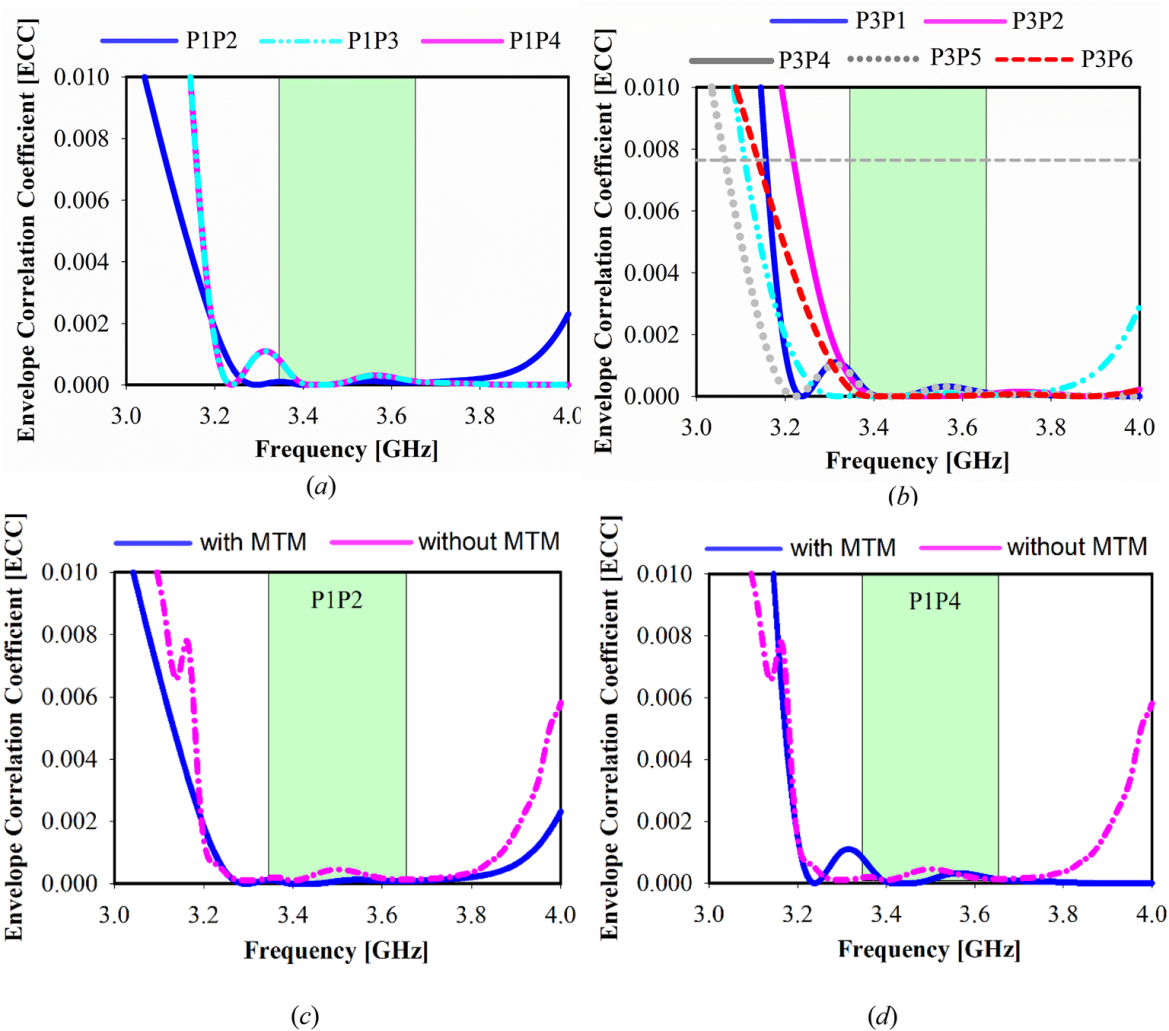
The Envelope correlation coefficient (ECC) of the suggested mMIMO antenna: (**a,b**) Port 1, 2, 3, 4, 5, 6, (**c,d**) Prot 1, 2, 4 with and without MTM.

The computed ECC of the proposed MIMO antenna array in the Sub 6 GHz bandwidth for 5G at 3.5 GHz is less than 0.0001, as illustrated in Fig. 15. As a result, the aforementioned result indicates that each of the two antenna ports has a low correlation, indicating great diversity performance. All ECCs are less than 0.0001, which meets the standard conditions of ECC is 0.3 in base station techniques due to high port-to-port isolation and the continuous radiation pattern. Moreover, Fig. 15c,d demonstrates the effect of MTM on the ECC results.

**Figure 16:**
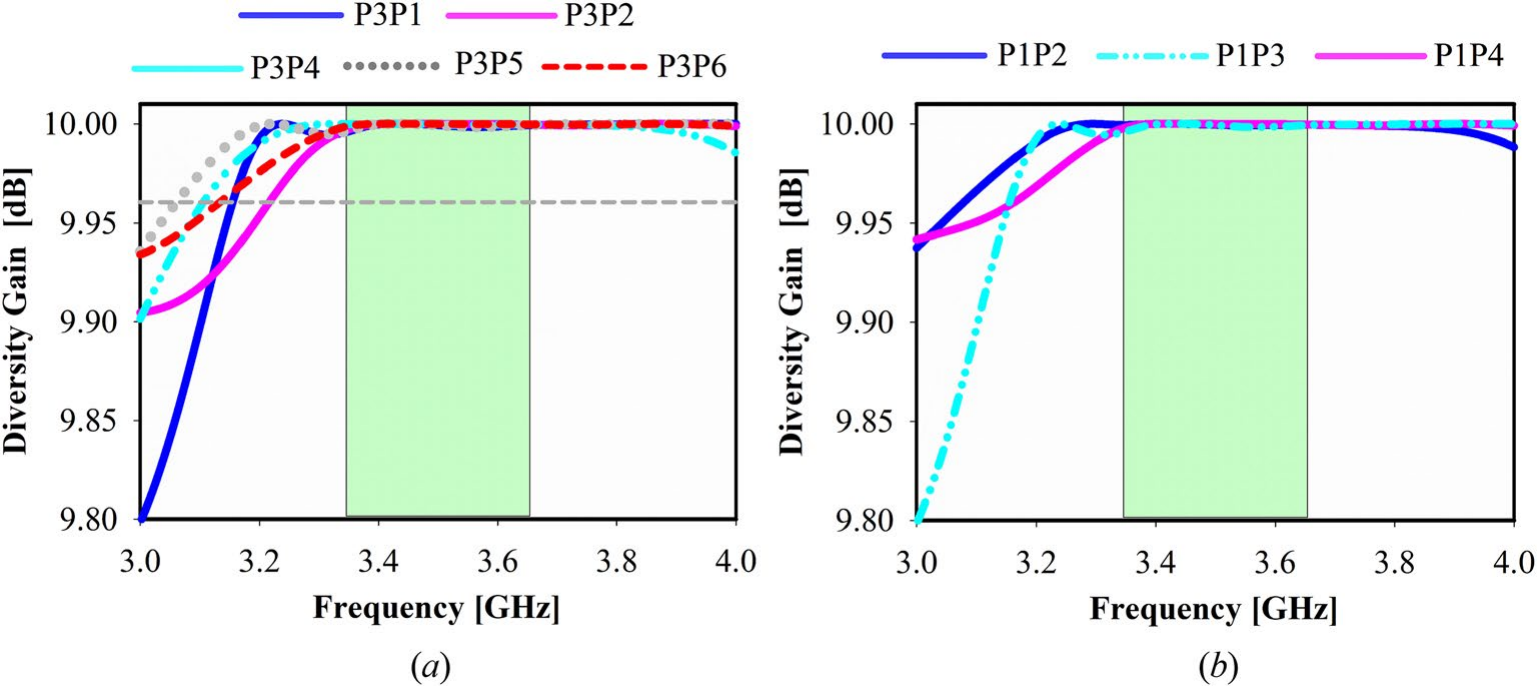
Diversity gain of the suggested mMIMO antenna, (**a**) Port 1, 2, 3, 4, 5, 6, (**b**) Prot 1, 2, 3, 4.

**Figure 17:**
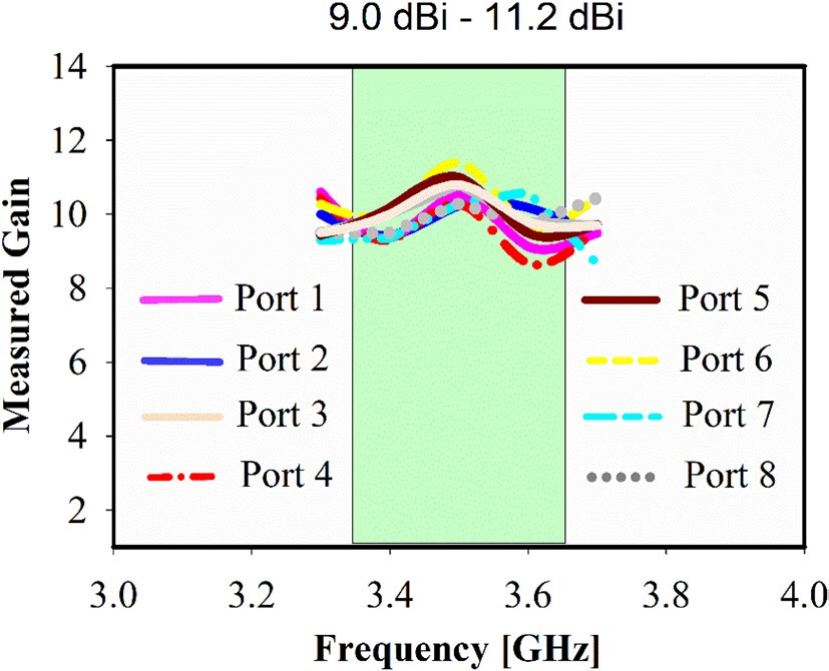
Gain of the mMIMO antenna: measured.

The diversity gain can be computed using $DG= 10 \times \sqrt{1- {ECC}^{2}}$, and the significant value of achieved diversity gain is 9.95 dB, as shown in Fig. 16a for port 1 to 4 and Fig. 16b for port 5 to 8. For optimal performance of mMIMO antenna diversity gain have to be near 10 dB. For various subarray ports, the measured realized gain is determined to be between 9.0 and 11.2 dBi within the band of interest, as illustrated in Fig. 17, which satisfies the functional requirements of base station application and makes the proposed antenna applicable for 5G communication. Besides high gain for each subarray, the realized broadside gain is 19.5 dBi.

**Figure 18:**
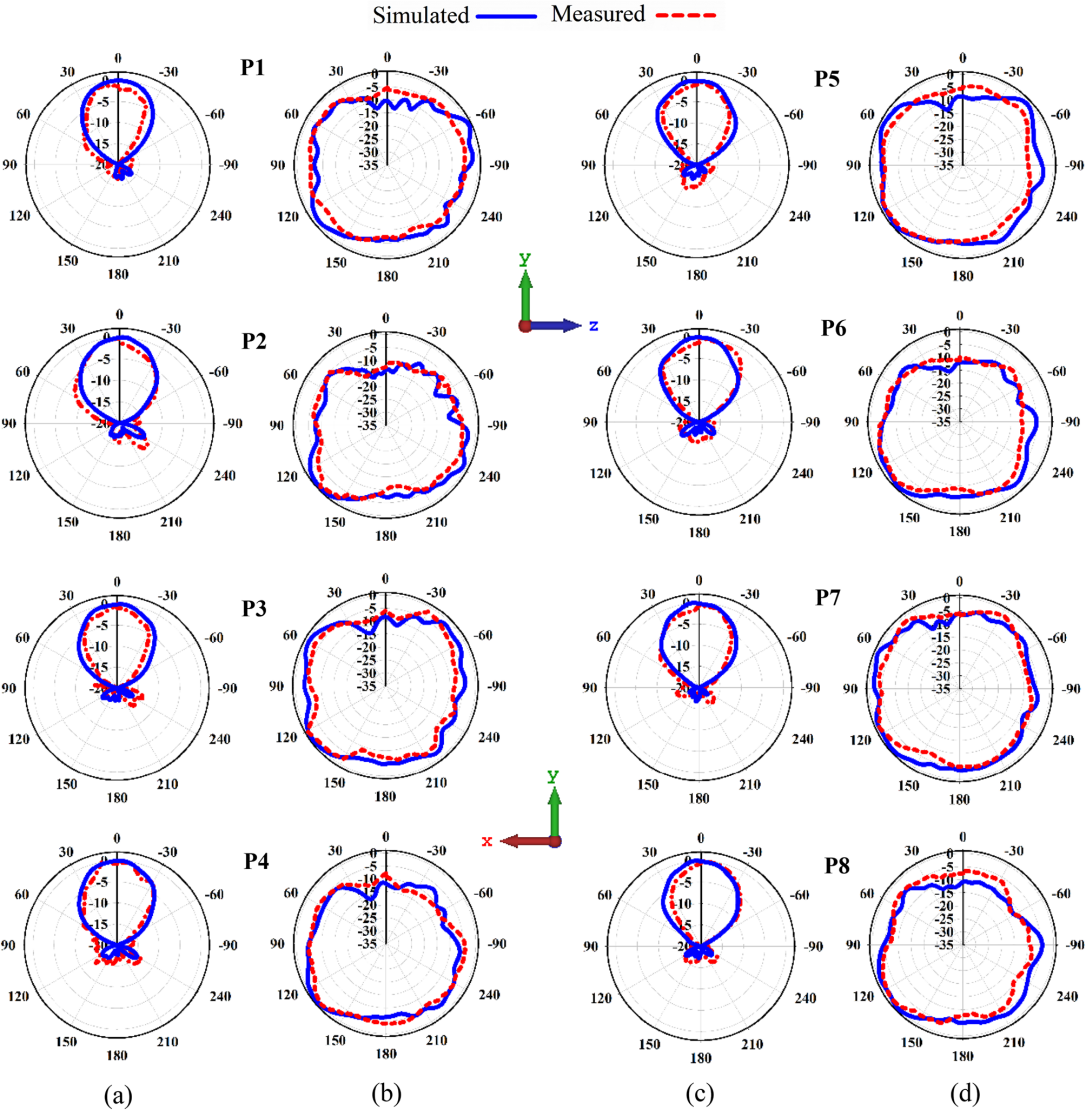
Normalized radiation patterns at 3.5 GHz for suggested planes at (**a,c**) YZ (Ø = $90^\circ$), (**b,d**) XY (θ = $90^\circ$): Simulated and measured.

**Figure 19:**
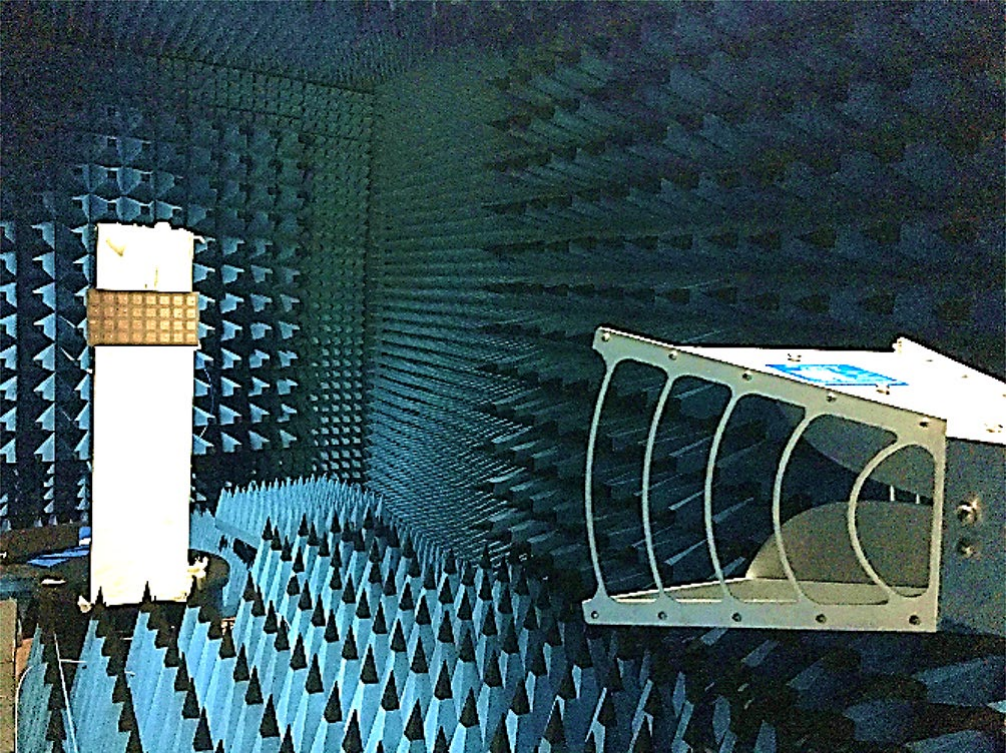
Setup of the radiation pattern for measurement.

Figure 18 depicts the proposed antenna elements’ normalised 3.5 GHz radiation patterns for each excited port. The co-polarization components are quite stable due to the isolation effect, and there are no visible ripples across the operating frequency band at 3.5 GHz. As illustrated in Fig. 18, the 2D radiation patterns of the proposed mMIMO antenna are measured and simulated in the E-plane at yz within $\mathrm{\varnothing }= 90^\circ$ whereas $\uptheta = 90^\circ$ in the xy direction (H-plane). The far-field properties indicate a superb directional broadside main beam at yz planes for port 1–8; when port 1 is excited, the other subarray ports become reflectors. However, a nearly omnidirectional occurs in xy-plane. In addition, the simulation and measurement results are noticeable in good agreement. Figure 19 shows the pattern measurement setup inside the Anchor Chamber.

## Conclusions

This paper presents a 32-element based mMIMO antenna system for 5G base stations that is integrated with an array of ENG/DNG metamaterial. The SSRR metamaterial unit cell is a one-of-a-kind symmetric split-ring resonator (SSRR) metamaterial unit cell. It has a large negative index with more than 1 GHz bandwidth for Epsilon-negative metamaterial (ENG) and the negative real value property of near-zero refractive index (NZRI) in the range of 3.1 GHz to 4.2 GHz for Epsilon-negative metamaterial (ENG).

Eight subarrays are placed on a single side panel with full and partial grounds, which are placed on the middle and backside of two layers, respectively. The minimum measured BW is achieved with 250 MHz, whereas the minimum measured gain is 9 dBi within the band of interest at 3.5 GHz. However, 90% is considered as the realized efficiency. Even when the two antennas are quite close to one another, high isolation can be achieved, whereas the maximum ECC performance between the ports is 0.0001. In conclusion, the suggested mMIMO array (with 32 compact elements) has shown good isolation and overall performance, making it a suitable contender for 5G sub-6 GHz base station applications.
